# Chloroplast genome sequence of the hybrid variety ‘Gagsi’, one of the colored calla lilies (*Zantedeschia* spp.)

**DOI:** 10.1080/23802359.2020.1827060

**Published:** 2020-10-05

**Authors:** Jong-Bo Kim, Hwan-Rae Yang, Sang-Hee Lee, Young-Jin Kim, Tae-Ho Park

**Affiliations:** a Department of Biotechnology, Research Institute for Biomedical & Health Sciences, College of Biomedical & Health Sciences, Glocal Campus, Konkuk University, Chungju, South Korea; b Horticultural Research Division, Gangwondo Agricultural Research and Extension Services, Chuncheon, South Korea; c Department of Horticulture, Daegu University, Gyeongsan, South Korea

**Keywords:** Chloroplast, genome, genome sequence, *Zantedeschia* hybrid

## Abstract

The complete chloroplast genome of *Zantedeschia* spp. in the family Araceae was constituted by *de novo* assembly using a small amount of whole genome sequencing data. The chloroplast genome of *Zantedeschia* spp. was the circular DNA molecule with a length of 175,448 bp and consisted of 90,244 bp of large single copy, 8334 bp of small single copy, and 38,435 bp of a pair of inverted repeat regions. A total of 163 genes were annotated including 109 protein-coding genes, 46 tRNA genes and eight rRNA genes. Maximum likelihood phylogenetic analysis with 16 Araceae species revealed that *Zantedeschia* spp. is expectedly grouped with other *Zantedeschia* species.

The *Zantedeschia* species belonging to the genus *Zantedeschia* and the family Araceae is a herbaceous perennial flowering plant originating from the swampy or mountainous regions of South Africa (Earl 1957). *Zantedeschia* spp. can be distinguished as two groups. The first group including *Z. aethiopica* and *Z. odorata* can be recognized by white flowers and the second group including *Z. albomaculata*, *Z. rehmannii*, *Z. elliottiana*, *Z. jucunda*, *Z. valida* and *Z. pentlandii* have colorful flowers (Funnell 1993; Singh et al. 1996; Kuehny 2000; Snijder et al. 2004). Interspecific hybrids between different species from the different two groups cannot be generated and developed in conventional breeding due to endosperm degeneration, abnormal embryo growth and arrested plastid development caused by plastome-genome incompatibility (Yao et al. 1994; Yao and Cohen 2000). Colored calla lilies referred to as *Zantedeschia* hybrids are interspecific hybrids derived from a cross between different species, mainly *Z. elliottiana*, *Z. pentlandii*, *Z. albomaculata* and *Z. rehmannii* and become one of the most popular horticultural crops worldwide (Funnell 1993; Snijder et al. 2004). However, The interspecific hybrids show chlorophyll deficiency causing more susceptible to soft rot than healthy green plants and biparental inheritance of plastid and plastome-genome incompatibility are prevalent in the interspecific hybrids (Snijder et al. 2004, 2007). The level of plastome-genome incompatibility depends on the relatedness of the parental species in the genus *Zantedeschia* (Snijder et al. 2007; Wei et al. 2017). Therefore, the information of chloroplast genome of the hybrid ‘Gagsi’ (*Zantedeschia* spp.) obtained in this study will provide an opportunity to breed colored calla efficiently and to investigate more detailed evolutionary aspect.

The plant of the colored calla lily variety ‘Gagsi’ was provided by Horticultural Research Division, Gangwondo Agricultural Research and Extension Services, South Korea (37.9°N, 127.7°E). The variety is a hybrid derived from a cross between the variety ‘Super gem’ (*Zantedeschia* spp.) and the variety ‘Best Gold’ (*Z. pentlandii*) and the color of the variety is variegated (light pink and yellow mixed) (Lee et al. 2018, 2019). An Illumina paired-end (PE) genomic library was constructed with a total genomic DNA according to the PE standard protocol (Illumina, San Diego, USA) and sequenced using an Illumina HiSeq2000 at Macrogen (http://www.macrogen.com/kor/). Low-quality bases with raw scores of 20 or less were removed and approximately 3.1 Gbp of high-quality of PE reads were assembled by a CLC genome assembler (CLC Inc, Rarhus, Denmark) (Kim et al. 2015). The reference chloroplast genome sequence of *Epipremnum aureum* (KR872391, Tian et al. 2018) was used to retrieve principal contigs representing the chloroplast genome from the total contigs using Nucmer (Kurtz et al. 2004). The representative chloroplast contigs were arranged in order based on BLASTZ analysis (Schwartz et al. 2003) with the reference sequence and connected to a single draft sequence by joining overlapping terminal sequences. DOGMA (Wyman et al. 2004) and BLAST searches were used to predict chloroplast genes.

The complete chloroplast genome of the *Zantedeschia* hybrid variety ‘Gagsi’ (GenBank accession no. MK327551) was 175,448 bp in length including 38,435 bp inverted repeats (IRa and IRb) regions separated by small single copy (SSC) region of 8334 bp and large single copy (LSC) region of 90,244 bp with the typical quadripartite structure of most plastids, and the structure and gene features were typically similar with those of higher plants. The total length was very similar with those of *Z. elliottinana* (175,906 bp) and *Z. rehmannii* (175,067 bp). However, it was longer than those of other species in the Araceae family. A total of 163 genes with an average size of 610.6 bp were annotated including 109 protein-coding genes with an average size of 803.1 bp, 46 tRNA genes, and 8 rRNA genes with an average size of 222.0 bp. An overall GC content was 35.60%.

Phylogenetic analysis was performed with 73 consensus coding sequences of the *Zantedeschia* spp. hybrid variety ‘Gagsi’ and 16 published species in the Araceae family by a maximum likelihood method in MEGA 6.0 (Tamura et al. 2013). According to the phylogenetic tree, interestingly the *Zantedeschia* hybrid variety ‘Gagsi’ was closely grouped with other colored calla lilies *Zantedeschia* species and *Epipremnum aureum* (Figure 1).

**Figure 1.**
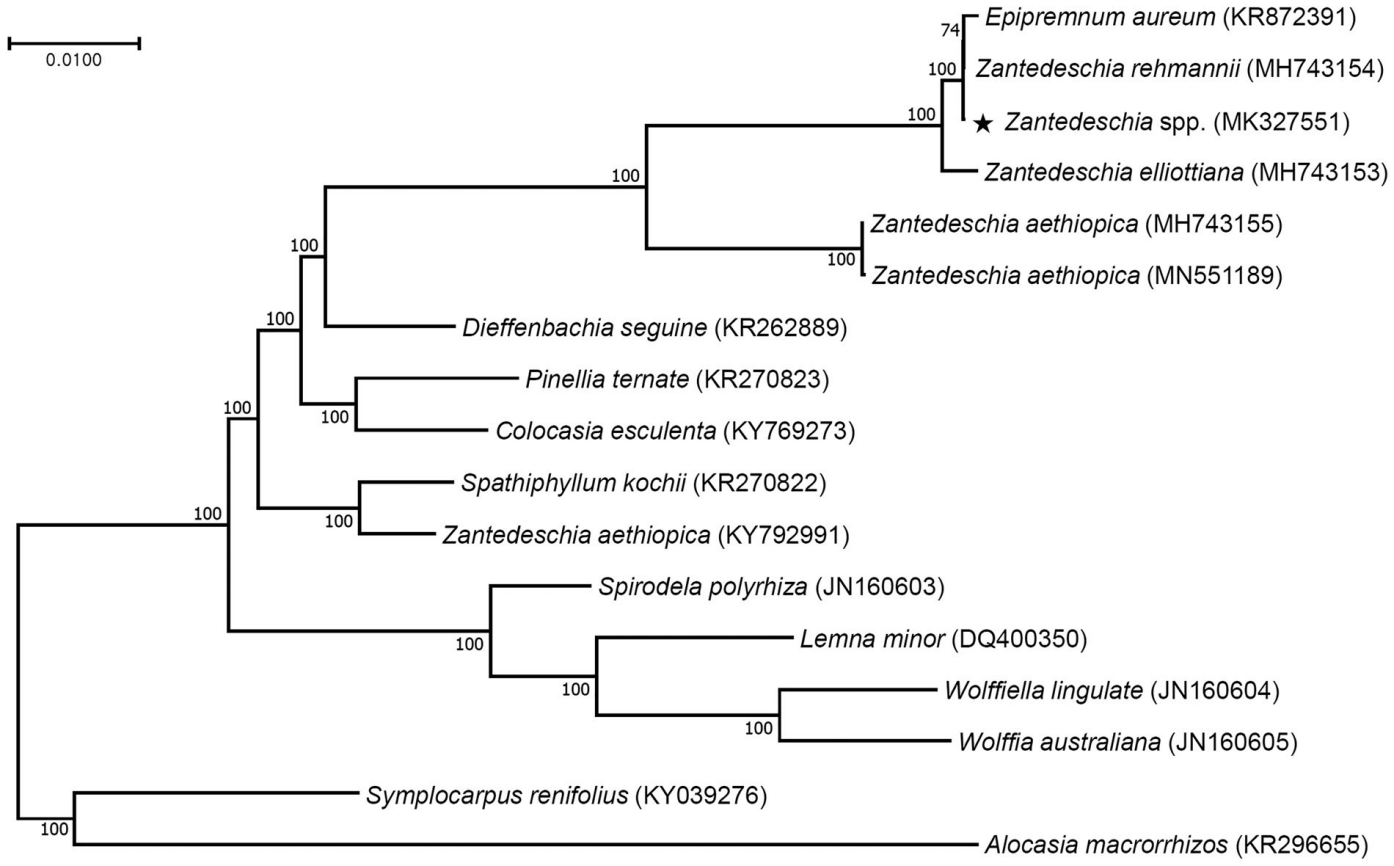
Maximum likelihood phylogenetic tree of *Zantedeschia* spp. with 16 species belonging to the Araceae family based on chloroplast protein coding sequences. Numbers in the nodes are the bootstrap values from 1000 replicates.

## Data Availability

The data that support the findings of this study are openly available in GenBank of NCBI at https://www.ncbi.nlm.nih.gov, reference number MK327551.
